# Apnoeic oxygenation by nasal cannula during airway management in children undergoing general anaesthesia: a pilot randomised controlled trial

**DOI:** 10.1186/s13741-018-0083-x

**Published:** 2018-02-21

**Authors:** Lafi Olayan, Abdulaleem Alatassi, Jaimin Patel, Sherran Milton

**Affiliations:** 1 College of Applied Medical Sciences, King Saud bin Abdulaziz University for Health Sciences, Riyadh, Saudi Arabia; 2 Department of Anesthesiology, King Abdulaziz Medical City, Riyadh, Saudi Arabia; 3 Institute of Inflammation and Ageing, University of Birmingham, Birmingham, UK; 4 School of Healthcare Sciences, Cardiff University, Cardiff, UK

**Keywords:** Apnoeic oxygenation, Airway management, Children, General anaesthesia

## Abstract

**Background:**

Airway management is a core clinical skill in anaesthesia. Pre-oxygenation prior to induction of anaesthesia is a standard practice to prevent desaturation. Apnoeic oxygenation in adults is effective and prolongs the time to desaturation. The effectiveness of apnoeic oxygenation in the adult is well documented; however, evidence in the paediatric is lacking. Therefore, the aim of this study was to investigate the effectiveness of apnoeic oxygenation during airway management in children.

**Methods:**

This was a pilot randomised controlled trial. Patients were randomised to receive either apnoeic oxygenation or standard care during the induction of anaesthesia. The primary outcome was the duration of safe apnoea, defined as a composite of the time to first event, either time for SpO2 to drop to 92% or time to successfully secure the airway, and the lowest SpO2 observed during airway management. Secondary outcomes were the number of patients whose SpO2 dropped below 95% and the number of patients whose SpO2 dropped below 92%.

**Results:**

A total of 30 patients were randomised, 15 to apnoeic oxygenation and 15 to standard care. No significant difference was observed in the time to first event (*p* = 0.870). However, patients randomised to apnoeic oxygenation had significantly higher SpO2 observed compared to the standard care group (*p* = 0.004). All patients in the apnoeic oxygenation group maintained SpO2 of 100% during airway management, compared to only six in the standard care group. SpO2 dropped below 92% in one patient, with the lowest SPO_2_ recorded 73%.

**Conclusion:**

This study suggests that providing 3 l/min oxygen by nasal cannula following pre-oxygenation contributes to maintaining high levels of oxygen saturation during airway management in children, contributing to increased patients’ safety during general anaesthesia.

**Trial registration:**

Retrospectively registered at ClinicalTrials.gov, NCT03271827. Registered: 4 September 2017.

## Background

Anaesthetic agents and muscle relaxants used in general anaesthesia inhibit spontaneous respiration, resulting in apnoea. During periods of apnoea, it is necessary to manage patients’ ventilation and oxygenation (Hughes and Mardell 2012). Therefore, airway management is an essential component within routine anaesthesia practice to manage patients’ ventilation and oxygenation (Al-Shaikh and Stacey 2007). During the intubation phase of airway management, patients are usually kept without ventilation and oxygenation until the airway is successfully secured (Wilkins et al. 2009). This is called the apnoeic period (Hughes and Mardell 2012). The time required to successfully secure the airway varies depending on the patency of the patient’s upper airways and the proficiency of the airway management provider (Sanders et al. 2013). Oxygen saturation gradually decreases as apnoea period during the airway management is prolonged (Wilkins et al. 2009). Providing sufficient oxygen before attempting to secure the airway, known as pre-oxygenation, is a standard oxygenation method that sustains adequate oxygen saturation during airway management by denitrogenising the lungs, providing an oxygen reservoir which facilitates extended intubation (Tanoubi et al. 2009).

Usually, the pre-oxygenation technique is the key factor that assures adequate oxygenation during airway management (Sirian and Wills 2009). However, providing continuous oxygenation during airway management, called apnoeic oxygenation, provides additional time for maintaining safe oxygen saturation. Apnoeic oxygenation is thought to be effective and worth considering in anaesthesiology. After pre-oxygenation, anaesthetised patients can still receive oxygen during intubation without hindering the laryngeal view. Consequently, apnoeic oxygenation may provide a longer margin of safety during airway management without significant desaturation, which allows extra time to secure the airway from the first attempt (Weingart and Levitan 2012).

In studies conducted by Baraka et al. (2007) and Ramachandran et al. (2010), the effectiveness of apnoeic oxygenation was tested on obese subjects in order to maintain safe apnoea time SpO2 > 95%. The apnoeic oxygenation used in Baraka et al. (2007) trial was 5 l/min oxygen insufflation via nasopharyngeal catheter. The apnoeic oxygenation was terminated if SpO2 fell below 95% or after 4 min had elapsed. Consequently, the results showed that apnoeic oxygenation is strongly effective in obese patients, as all patients of the study group maintained 100% SpO2 during the 4-min period. Where patients did not receive apnoeic oxygenation, their SpO2 dropped below 95% within 145 s. Ramachandran et al. (2010) considered that 4 min of safe apnoea, which is used by Baraka et al. (2007), is a too short time period to measure the effectiveness of apnoeic oxygenation on obese patients. Therefore, Ramachandran et al. (2010) tested apnoeic oxygenation by measuring safe apnoea duration (SpO2 > 95%) for 6 min. In addition, nasal prongs were used to deliver 5 l/min oxygen instead of nasopharyngeal catheters. The results suggested that apnoeic oxygenation via 5 l/min nasal prongs is also able to sustain SpO2 > 95% for 6 min. Furthermore, some hospitals considered apnoeic oxygenation within their standard protocols of airway management in intra-hospital and out-of-hospital emergency departments (Wimalasena et al. 2015). In a retrospective study, the rate of desaturation during emergency intubation was successfully decreased after standardising the use of apnoeic oxygenation via a 5 l/min nasal cannula (Wimalasena et al. 2015). In addition, the success rate of the first intubation attempts was increased after the introduction of apnoeic oxygenation.

The evidence has recommended that apnoeic oxygenation be considered in addition to pre-oxygenation, which contributes to changing airway management practice (Wimalasena et al. 2015). However, none of the studies addressed the effectiveness of apnoeic oxygenation on children, leaving a gap in the anaesthesia literature. Therefore, the aim of this study is to investigate the effectiveness of apnoeic oxygenation via nasal cannula for children, whose safe apnoea time is very short.

## Methods

A pilot randomised controlled trial design was used in this study which is part of a student project at Cardiff University. The study was conducted in the anaesthesia department of King Abdullah Specialist Children’s Hospital, Riyadh, Saudi Arabia.

A total of 102 patients were screened for their eligibility to be included in the study at a pre-anaesthesia clinic over a period of 4 weeks. A total of 30 patients, consisting of 11 females and 19 males, were recruited for analysis. All subjects were screened for their suitability by anaesthetists and the researcher based on the inclusion and exclusion criteria (Table 1).

**Table 1:** Inclusion and exclusion criteria

Inclusion	- Patients who were scheduled for elective surgery under general anaesthesia were included.
	- Age: one to 8 years old.
	- ASA I and II only without mild respiratory diseases.
	- Patients with normal cardiorespiratory function.
Exclusion	- Children undergoing dental surgeries in which nasal intubation is needed.
	- Patients who suffered from quick drops in oxygen saturation more rapidly than healthy children due to different reasons such as respiratory and pulmonary diseases, asthma, active or recent upper respiratory tract infection, syndromes with cardiopulmonary pathologies, cardiac anomalies, anaemia, depressed respiratory effort, ventilation/perfusion imbalance, obstructive sleep apnoea (OSA), and airway obstruction.
	- Patients reported with nasal obstruction.
	- Patients with grades of laryngoscopic view (Cormack-Lehane) greater than II, which indicates upper airway obstruction.

After gaining parental consent, the participants were randomly allocated to the apnoeic oxygenation (intervention) group, who received oxygen via a nasal cannula during airway management, or the standard care (control) group, who received standard airway management. The randomisation was done using sealed envelopes by requesting patients’ parents to pull out one envelope at random. The envelopes were handed over to parents by the anaesthetist, who was an independent investigator.

**Fig. 1:**
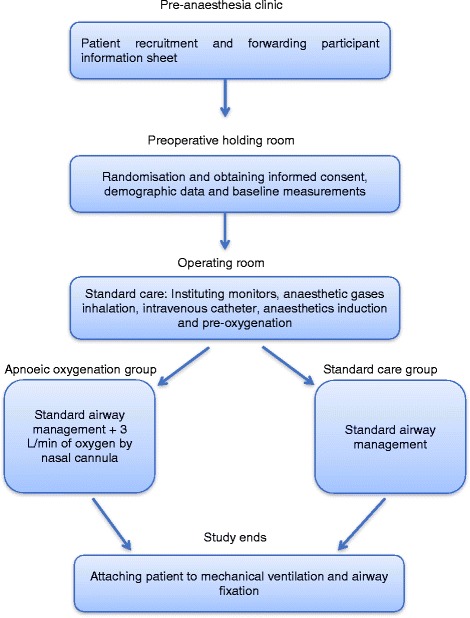
Flow chart to demonstrate the study protocol

The 15 patients assigned to the apnoeic oxygenation group received low flow of oxygen (3 l/min) via nasal cannula (SOF-TOUCH nasal cannula, Unomedical, Canva Tec Limited, Flintshire, UK) during the apnoea period, while the other 15 patients received no oxygen via nasal cannula during apnoea. The apnoea period is the time spent on airway insertion, which is measured from the end of pre-oxygenation, after mask ventilation, until the airway is successfully secured and mechanical ventilation is commenced (Weingart and Levitan 2012). Successful securing of the airway, where the apnoeic period ends, was ensured by observing the first chest movement and getting positive end-tidal CO_2_ readings. The nasal cannula was connected to an external oxygen regulator incorporated into the anaesthesia machine (Infinity C500, Draeger Medical Systems, PA, USA). All patients in both groups received standard care according to the hospital’s guidelines, which follow the American Society of Anesthesiologists (ASA) guidelines for paediatric anaesthesia, as in the following flow diagram (Fig. 1):

The primary outcome measure in this study was the duration of safe apnoea. Safe apnoea time was defined as the period of time when SpO2 was maintained above 92%, where critical hypoxemia occurs during apnoea (Wilkins et al. 2009; Ramachandran et al. 2010). The duration of safe apnoea is measured by allowing desaturation during apnoea that continued until SpO2 dropped to 95% or until a 6-min cut-off time had elapsed (Taha et al. 2006). However, the cut-off time, which allows desaturation, was not used in this study for ethical reasons. Instead, the primary outcome of this study was measured by time to first event: either time for SpO2 to fall to 92% or time to successfully secure the airway as usual practice without allowing desaturation. In addition, the lowest SpO2 observed during airway management was obtained to compare the two groups.

Secondary outcomes in this study were the number of patients whose SpO2 dropped below 95% and the number of patients whose SpO2 dropped below 92%. The SpO2 was measured using a pulse oximeter (Infinity Delta, Draeger Medical Systems, PA, USA) incorporated in the anaesthesia machine (Infinity C500, Draeger Medical Systems, PA, USA). The pulse oximeter was placed on the patient’s finger, which is considered a valid and reliable measurement of oxygen saturation in children (Bilan et al. 2010). The reliability of the pulse oximeter was ensured by calibration of the anaesthesia machine according to the manufacturer’s specifications.

Statistical analysis was performed using Statistical Package for the Social Sciences (SPSS) version 20 (IBM Corp., Armonk, NY). Efficacy analysis was executed based on the principle of intent to treat (ITT). All subjects were analysed in their assigned groups, originally allocated by randomisation, regardless of whether they dropped out or there was any deviation from protocol.

Interval variables were analysed using *T* test for normally distributed data and Mann-Whitney *U* test for non-normally distributed data. Results were reported as mean, standard error and corresponding *p* value for normally distributed data and median, interquartile range (IQR) and *p* value for non-normally distributed data. Categorical variables, including the secondary outcomes: the number of patients whose SpO2 dropped below 95% and the number of patients whose SpO2 dropped below 92%, were analysed using chi-square/Fisher’s exact test. Results were reported as counts, percentages and corresponding *p* values. Significance was declared at alpha less than 0.05.

According to Julious (2005), a minimum of 12 participants per group are needed for pilot trials based on gains in precision about the mean and variance (Figure 3.1). Accordingly, a sample size of 15 patients per group was appropriate for this preliminary study, with a total of 30 patients.

## Results

**Fig. 2:**
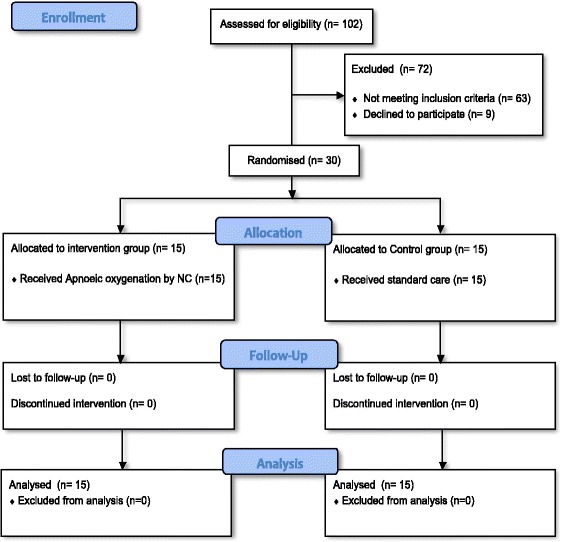
Flow diagram of participants’ enrolment

A total of 102 patients were screened for eligibility based on the inclusion and exclusion criteria. Sixty-three patients did not meet the eligibility criteria and nine patients declined to have their children participate in the study. Subsequently, 30 patients were recruited for this study and all patients were included in the analysis, as no dropout or discontinuation of the intervention was encountered (Fig. 2).

Chi-square/Fisher’s exact test for categorical variables revealed no significant difference in gender (*p* = 0.256) and ASA class (*p* = 1.000) and significant difference in surgery type (*p* = 0.022) between the two groups. *T* test and Mann-Whitney *U* test as appropriate based on normality tests. The results of the *T* test and Mann-Whitney *U* test showed no significant difference between the two groups in age (*T* = 0.473, *p* = 0.640), height (*T* = 0.971, *p* = 0.340) and weight (*U* = 85, *p* = 0.267) (Table 2). The Mann-Whitney *U* test showed no significant difference between the two groups in HR (*U* = 98.5, *p* = 0.567), MAP (*U* = 105.5, *p* = 0.775) and SpO2 (*U* = 108, *p* = 0. 0.870) at baseline (Table 3). Accordingly, the balance of all demographics, except for surgery type, and baseline clinical characteristics was maintained between the control and intervention groups before starting the intervention.

**Table 2:** T-test, Mann-Whitney U test and Chi-Square/Fisher’s Exact test for all demographics

Variable	Control group	Intervention group	T-test P-value
	Mean ± SD		
Age (m)	56.80 ± 24.487	52.40 ± 26.416	T= 0.473
			p=0.640
Height (cm)	111.53 ± 13.89	106.27 ± 15.764	T= 0.971
			p=0.340
	Median (IQR)		U Value
			P-value
Weight (kg)	21 (14 - 23)	14 (13 - 22)	U= 85
			p=0.267
	Count (%)		Chi-square
			P-value
Gender			1.292
Male: Female	8 (53.3%): 7 (46.7%)	11(73.3%): 4 (26.7%)	p=0.256
ASA class			
ASA I	12 (80%)	11 (73.3%)	0.187
ASA II	3 (20%)	4 (26.7%)	p=1.000^a^
Surgery type			
General surgery:	4 (26.7%)	10 (66.7%)	
ENT surgery:	5 (33.3%)	3 (20%)	
Plastic surgery:	1 (6.7%)	1 (6.7%)	
Ophthalmology surgery:	5 (33.3%)	0 (0%)	11.480
Neurosurgery:	0 (0%)	1 (6.7%)	p=0.022^b,c^

M = Month, cm = centimetre, kg = kilogram, ASA class = American Society of Anesthesiologists classification, IQR= Interquartile Range

^a^ Fisher’s Exact is used instead of Pearson Chi-Square when an assumption is violated

^b^ Likelihood ratio is used instead of Pearson Chi-Square when an assumption is violated and the table is bigger than 2*2

^c^ P-value < 0.05 indicates a significant difference between the groups

**Table 3:** Baseline clinical characteristics between the two groups

Variable	Median (IQR)		U Value
	Control group	Intervention group	P-value
HR (bpm)	101 (90–116)	107 (95–111)	U = 98.5
			p = 0.567
MAP (mmHg)	77 (66–82)	73 (69–82)	U = 105.5
			p = 0.775
SpO2_Baseline (%)	100 (99–100)	100 (99–100)	U = 108
			p = 0.870

HR = Heart Rate, bpm = Beat Per Minute, MAP = Mean Arterial Pressure, mmHg = millimetres of mercury, SpO2_Baseline = SpO2 at baseline, IQR = Interquartile Range

Table 4 presents the result of Mann-Whitney *U* test for the primary outcomes, time to first event and lowest SpO2 observed during airway management. The median time to first event in the control group was not significantly higher than the median in the intervention group (median (IQR) of control vs. intervention; 39 (19–64) vs. 34 (21–55), *U* = 108.5, *p* = 0.870). On the other hand, the control group did not maintain a lowest SpO2 as high as the intervention group (median (IQR) of control vs. intervention; 99 (98–100) vs. 100 (100–100), *U* = 45, *p* = 0.870).

**Table 4:** Mann-Whitney U for T_first and SpO2_Lowest

Variable	Median (IQR)		Mann-Whitney U
	Control group	Intervention group	P-value
T_first (sec)	39 (19–64)	34 (21–55)	U = 108.5
			p = 0.870
SpO2_Lowest (%)	99 (98–100)	100 (100–100)	U = 45
			p = 0.004*

T_first = Time to first event, sec = Seconds, SpO2_Lowest = lowest SpO2 observed during airway management, IQR = Interquartile Range

*P-value < 0.05, indicating a significant difference between the groups

Patients in the control group reached different levels of SpO2 below 100% during airway management. On the other hand, all patients in the intervention group maintained 100% SpO2, whereas only six (40%) patients in the control group, who did not receive apnoeic oxygenation, maintained 100% SpO2. In addition, one patient in the control group reached SpO2 of 73%, whereas the other patients in the control group maintained SpO2 of more than 95% but not 100% (*p* = 0.002) (Table 5).

**Table 5:** Difference in lowest SpO2 levels between the two groups

SpO2%	Count (Percentage)		Chi-square P-value
	Control group	Intervention group	
SpO2 73%	1 (6.7%)	0 (0%)	16.462 p = 0.002 ^a,b^
SpO2 96%	2 (13.3%)	0 (0%)	
SpO2 98%	4 (26.7%)	0 (0%)	
SpO2 99%	2 (13.3%)	0 (0%)	
SpO2 100%	6 (40%)	15 (100%)	
Total	15 (100%)	15 (100%)	

SpO2% = Percentage of Oxygen Saturation, N = Number of patients

^a^Likelihood ratio is used instead of Pearson Chi-Square when an assumption is violated and the table is bigger than 2*2

^b^P-value < 0.05 which indicates a significant difference between the groups

## Discussion

The aim of this pilot study was to investigate the effectiveness of apnoeic oxygenation via nasal cannula in a paediatric population. The effectiveness of apnoeic oxygenation was evaluated by measuring two primary outcomes: duration of safe apnoea and lowest SpO2 observed during airway management. In this study, the definition of the duration of safe apnoea was based on time to first event: either time for SpO2 to drop to 92% or time to successfully secure the airways without allowing desaturation. Accordingly, there was no difference in the duration of safe apnoea between the apnoeic oxygenation group and the standard care group in this pilot study. On the other hand, the lowest SpO2 in the apnoeic oxygenation group was significantly higher than that in the standard care group, who did not receive apnoeic oxygenation during airway management.

There is a paucity of anaesthesia literature investigating the effectiveness of apnoeic oxygenation in children, whereas the majority of the literature is directed at the adult population. It was therefore considered necessary to discuss the effectiveness of apnoeic oxygenation on adults in the literature review. However, the benefits of apnoeic oxygenation for children might be considered equally important, if not more so, as children are normally prone to hypoxemia and have shorter duration of safe apnoea (SpO2 > 92%) during airway management, owing to their physiological differences (Sirian and Wills 2009). The effects of these physiological differences in paediatric patients, including functional residual capacity (FRC) and oxygen consumption rate, contribute to the critical changes in the duration of safe apnoea, as illustrated by Hardman et al. (2000). The prolongation of safe apnoea duration for paediatric patients is crucial during airway management and provides sufficient time for anaesthetists or other airway management practitioners to secure the airway without a critical drop in oxygen saturation. The present study attempted to extend the duration of safe apnoea (SpO2 > 92%) for an extended period of time by applying 3 l/min nasal cannula as an apnoeic oxygenation technique in addition to pre-oxygenation with tidal volume breathing of 100% oxygen for 3 min. The results did not show a significant difference in the duration of safe apnoea between patients who received apnoeic oxygenation with pre-oxygenation and patients who received pre-oxygenation alone. This suggests that apnoeic oxygenation is not effective in prolonging the safe apnoea time. However, this study defined safe apnoea duration as time to first event: either time for oxygen saturation to drop from 100 to 92% or time to successfully secure the airway, without using cut-off time and allowing desaturation. Consequently, all patients in this study did not reach oxygen saturation of 92%, except for one patient whose SpO2 reached 73%. This means that the duration of safe apnoea in this study did not exactly reflect the time for SpO2 to drop to 92% due to ethical considerations, as allowing desaturation is extremely unsafe for children, so the use of cut-off time was not feasible in this study.

Although the results showed that apnoeic oxygenation was not able to provide a longer duration of safe apnoea in this study, the lowest oxygen saturation observed was significantly different between the two groups. The result suggests that the lowest oxygen saturation in the apnoeic oxygenation group is higher than that in the standard care group, but did not show the duration of sustaining this high level of oxygen saturation, represented by the duration of safe apnoea. The lowest SpO2 results suggested that apnoeic oxygenation is effective in children when using 3 l/min oxygen via nasal cannula after pre-oxygenation with 100% oxygen for 3 min. All patients who received apnoeic oxygenation following pre-oxygenation sustained SpO2 of 100% during airway management. Conversely, only six of the 15 patients who received pre-oxygenation alone maintained SpO2 of 100%. In addition, one patient in the standard care group experienced desaturation to SpO2 of 73%, while the other patients in this group maintained normal oxygen saturation, more than 95%, but not 100%. The results for the lowest SpO2 observed are promising in terms of the effectiveness of apnoeic oxygenation in the paediatric population.

It must be noted that the pre-oxygenation technique contributes to maintaining high oxygen saturation levels in addition to apnoeic oxygenation (Sirian and Wills 2009). In this study, pre-oxygenation was achieved by tidal volume breathing (TVB) with 100% oxygen for 3 min. The pre-oxygenation technique used is considered a very effective method of pre-oxygenation in most populations, including paediatric patients (Tanoubi et al. 2009; Hardman and Wills 2006; Chiron et al. 2007). For this reason, the pre-oxygenation technique used in this study contributed in sustaining oxygen saturation of 100% in the six patients who received pre-oxygenation alone.

This study found that only one patient experienced desaturation below SpO2 of 92%, while the other 14 patients in the same group, which was a standard care group, maintained normal oxygen saturation. There might be a few reasons to explain this desaturation other than not applying apnoeic oxygenation. First, patients’ clinical status might affect oxygenation or contribute to increased oxygen consumption rate, such as cardiopulmonary pathologies or recent infection (Murray and Nadel 2010). However, all patients in both groups were healthy children and were free of any cardiorespiratory disease, cardiac anomalies or any clinical status that affects oxygenation, as only patients of ASA class I and class II were included in this study. Second, differences in demographics, including age, gender, height and weight, and baseline SpO2 between the two groups might also affect the result. Nevertheless, all patients’ demographics and baseline clinical characteristics were not significantly different between the two groups, with the exception of surgery type, which is deemed less important. Accordingly, the balance between the two groups was maintained, which helps in controlling confounding factors and maintaining high internal validity (Hicks 2009). Third, the reliability of the pulse oximeter used might affect the detection of hypoxemia. However, the pulse oximeter probe was placed on the finger, which is reliable and valid in measuring oxygen saturation and correlates very closely to arterial blood gas results (Bilan et al. 2010). In addition, the pulse oximeter was calibrated daily in order to ensure its reliability.

Consideration has to be given to the time taken to secure the airway. Securing the airway takes time and helps to further reduce oxygen saturation, since the time taken to secure the airway is inversely proportional to oxygen saturation (Xue et al. 1996). In other words, oxygen saturation decreases if securing the airway is prolonged. In this study, the duration of safe apnoea, which reflects the time to secure the airway, was not significantly different between the two groups. Additionally, the return to spontaneous breathing during airway management was minimised by using appropriate anaesthesia medications and doses during anaesthetic induction (Singh and Frenkel 2013). Hence, all patients in both groups received appropriate doses of propofol, which is a standard anaesthetic drug, and rocuronium, which is a muscle relaxant that inhibits spontaneous breathing and facilitates airway management. It is thought that succinylcholine, which is a muscle relaxant, contributes to decreasing oxygen saturation during apnoea, as it increases oxygen consumption during depolarisation when fasciculations are evident (Singh and Frenkel 2013). For this reason, rocuronium was used in this study rather than succinylcholine, as rocuronium is the recommended and standard muscle relaxant in paediatric anaesthesiology.

Consequently, all potential factors discussed above that negatively impact the true effect of apnoeic oxygenation were minimised and controlled appropriately. As hypothesised in this study, apnoeic oxygenation via nasal cannula in children successfully maintained SpO2 100% during the median time of 34 s with an interquartile range of 21–55 s.

There are some limitations that need to be taken into account when interpreting the findings of this study. The chosen sample size for this pilot trial was based on recommendations for pilot studies from the literature (Johanson and Brooks 2009). Previous research exploring the effectiveness of apnoeic oxygenation in paediatric patients is lacking in the literature. It is, however, acknowledged that the low sample size might limit the power of the present findings, and therefore, the true effectiveness of apnoeic oxygenation in children is liable to type I and type II errors (Bryman 2012). As a result of this, the present study showed only preliminary and promising but not conclusive results. The findings might also lack generalisability, which is a common threat for most RCTs, as a result of the strict inclusion criteria used for the study (Sanson-Fisher et al. 2007). However, this study aimed to assess the effectiveness of the intervention on healthy children to eliminate any confounding factors affecting oxygen saturation, such as respiratory diseases and cardiopulmonary pathologies. Patients’ demographics chosen for this study contribute to limiting the generalisability of the results to the paediatric population.

Previous researches measured the duration of safe apnoea by using a cut-off time in which desaturation was allowed in order to measure the exact duration of safe apnoea. However, as mentioned above, this could be difficult to achieve in children, who are very sensitive to hypoxemia (Hardman and Wills 2006). Alternatively, time to first event—either time for SpO2 to drop to 92% or time to successfully secure the airway—was measured in order to avoid any critical drop in oxygen saturation caused by the trial. Thus, it must be acknowledged that time to first event does not exactly reflect the duration of safe apnoea. It must be taken into account that the time to successfully secure the airway is variable and depends on many factors, including the type and size of the tube and the provider’s skills and proficiency (Wilkins et al. 2009; Sanders et al. 2013). In this study, airway management was accomplished by both anaesthetists (12 in intervention group and 10 in control group) and less expert providers, including junior residents (one in control group) and respiratory therapy trainees (three in intervention group and four in control group). Although the variation in providers’ proficiency was not controlled in this study, the time to secure the airway was not statistically different between the two groups. Therefore, the result of the lowest oxygen saturation observed was not biased by the levels of proficiency between the different providers in the two groups.

The outcome assessor was not blinded during data collection. However, patients and researcher were not aware of patient group allocation until the intervention started, when further blinding was not possible due to the nature of the intervention (Ramachandran et al. 2010). In addition, patients were kept unaware of their group allocation during the intervention, since they were under general anaesthesia.

Further research into the effectiveness of apnoeic oxygenation on children should address the limitations of this pilot study. A large-scale randomised controlled trial would provide better findings and more conclusive results. Previous studies on healthy adults and obese patients have measured the duration of safe apnoea and the lowest oxygen saturation observed by allowing desaturation. Although allowing desaturation is unsafe in children, it might be appropriate for future researches to use cut-off time for a period of time, but not as long as the cut-off time used in adults, who tolerate desaturation for longer than children. Alternatively, it would be more appropriate to use simulators to investigate such hypotheses, which might threaten patients’ safety, by modelling investigations for different age groups in the paediatric population. Although in vivo research offers a conclusive insight into the effectiveness of an intervention, valid simulators are still effective and would provide useful results, especially when the data is difficult to obtain for ethical reasons (Hardman and Wills 2006). In addition, modelling investigations would allow in-depth exploration of the effectiveness of apnoeic oxygenation when simulating children by investigating different scenarios, including opened and closed airways, as illustrated by Hardman and Wills (2006) when investigating pre-oxygenation in children.

The present findings of this study would improve the current knowledge and clinical practice of paediatric airway management. Providing 3 l/min oxygen by nasal cannula following pre-oxygenation contributes to maintaining high levels of oxygen saturation during airway management in children. Consequently, apnoeic oxygenation would provide continuous oxygenation in apnoeic children, which therefore contributes in enhancing patients’ safety during general anaesthesia. Apnoeic oxygenation for adults is now being considered within the standard airway management protocol in some hospitals (Wimalasena et al. 2015).

Anaesthetists and other airway management providers are always careful when intubating children, since oxygen saturation in children is normally maintained for a short period of time (Sanders et al. 2013; Hardman et al. 2000). Multiple intubation attempts would be expected more in children and neonates as a result of early desaturation, which requires patients’ oxygenation to be restabilised (Sanders et al. 2013). For this reason, there are limited opportunities for novice practitioners and trainees to practice airway management in children because they need extended time to successfully secure the airway (Sanders et al. 2013). Application of apnoeic oxygenation would maintain oxygen saturation, as suggested by the results of this study, and would also provide longer duration of safe apnoea, as suggested by the previous studies on adults illustrated by Taha et al. (2006) and Ramachandran et al. (2010). As a result, additional safe apnoea time provided by apnoeic oxygenation would provide greater opportunities for novice practitioners to take part in paediatric airway management, which would enhance training level and clinical skills, especially in teaching hospitals.

## Conclusion

This pilot randomised controlled trial attempted to investigating the effectiveness of apnoeic oxygenation, by 3 l/min nasal cannula, on children undergoing general anaesthesia. The results showed no significant difference between the two groups on duration of safe apnoea, which contradicts previous studies on healthy adults and obese patients due to methodological differences. However, the lowest oxygen saturation observed was statistically different between the two groups. The results in terms of the lowest oxygen saturation observed in this study are consistent with previous studies on adults, suggesting that apnoeic oxygenation is an effective method of oxygenation to maintain high oxygen saturation during airway management. A large-scale randomised controlled trial would confirm the present findings, as the low sample size and duration of this study limit its rigour.

## Abbreviations

ASA: American Society of Anesthesiologists
HR: Heart rate
IQR: Interquartile range
ITT: Intent to treat
MAP: Mean arterial pressure
OSA: Obstructive sleep apnoea
SD: Standard deviation
SpO2_Lowest: Lowest SpO2 observed during airway management
SPSS: Statistical Package for the Social Sciences
T_first: Time to first event
